# Two New Dihydrosphingosine Analogs Against *Mycobacterium tuberculosis* Affect *gltA1*, *lprQ, and rpsO* Expression

**DOI:** 10.3389/fmicb.2021.742867

**Published:** 2021-11-03

**Authors:** Katia Peñuelas-Urquides, Mario Bermúdez de León, Beatriz Silva-Ramírez, Fabiola Castorena-Torres, Gloria María Molina-Salinas, Jorge Castro-Garza, Pola Becerril-Montes, Esther del Olmo, Arturo San Feliciano, Laura Adiene González-Escalante, Licet Villarreal-Treviño, Salvador Said-Fernández

**Affiliations:** ^1^Departamento de Biología Molecular, Centro de Investigación Biomédica del Noreste, Instituto Mexicano del Seguro Social, Monterrey, Mexico; ^2^Facultad de Ciencias Biológicas, Universidad Autónoma de Nuevo León (UANL), San Nicolás de los Garza, Mexico; ^3^Departamento de Inmunogenética, Centro de Investigación Biomédica del Noreste, Instituto Mexicano del Seguro Social, Monterrey, Mexico; ^4^Tecnologico de Monterrey, Escuela de Medicina, Monterrey, Mexico; ^5^Unidad de Investigación Médica Yucatán, Unidad Médica de Alta Especialidad, Hospital de Especialidades Centro Médico Nacional Ignacio García Téllez, Instituto Mexicano del Seguro Social, Mérida, Mexico; ^6^Laboratorio de Patogénesis Molecular, Centro de Investigación Biomédica del Noreste, Instituto Mexicano del Seguro Social, Monterrey, Mexico; ^7^Departamento de Biología Celular, Centro de Investigación Biomédica del Noreste, Instituto Mexicano del Seguro Social, Monterrey, Mexico; ^8^Departamento de Ciencias Farmacéuticas, Área de Química Farmacéutica, Facultad de Farmacia, Centro de Enfermedades Tropicales de la Universidad de Salamanca (CIETUS), Instituto de Investigación Biomédica de Salamanca (IBSAL), Universidad de Salamanca, Salamanca, Spain; ^9^Departamento de Bioquímica y Medicina Molecular, Facultad de Medicina, Universidad Autónoma de Nuevo León (UANL), Monterrey, Mexico

**Keywords:** *Mycobacterium tuberculosis*, multidrug-resistance, gene expression, antitubercular drugs, dihydrosphingosine analogs

## Abstract

The emergence of multidrug-resistant (MDR) *Mycobacterium tuberculosis* strains threaten the control of tuberculosis. New antitubercular dihydrosphingosine analogs, named UCIs, have been evaluated in preclinical studies but their cellular and molecular mechanisms of action against *M. tuberculosis* are still unknown. The aim of this study was to evaluate the effect of UCI exposure on gene expression of drug-sensitive H37Rv and MDR CIBIN:UMF:15:99 clones of *M. tuberculosis* which were isolated, phenotypically, and genetically characterized, cultured to log phase and treated with UCI compounds; followed by total RNA isolation, reverse transcription and hybridization assays on Affymetrix genomic microarrays. Data were validated with RT-qPCR assays. As results, UCI-05 and UCI-14 exposure increased *gltA1* expression in drug-sensitive H37Rv clones. Furthermore, UCI-05 increased *lprQ* expression in MDR CIBIN:UMF:15:99 *M. tuberculosis* clones while UCI-14 reduced the expression of this gene in drug-sensitive H37Rv clones. In addition, UCI-05 reduced *rpsO* expression in drug-sensitive H37Rv clones. We found gene expression alterations that suggest these molecules may alter carbon and lipid metabolism as well as interfere in the protein-producing machinery in *M. tuberculosis*.

## Introduction

Tuberculosis (TB) is caused primarily by *Mycobacterium tuberculosis* and is one of the leading causes of death attributable to a single infectious agent. In 2019 it was estimated that 10.0 million people developed TB (WHO, 2020). The emergence of drug-resistant, multidrug-resistant (MDR) and extensively drug-resistant strains has complicated the control of this disease. The development of new antitubercular drugs is essential to limit morbidity, mortality and transmission of these strains.

**FIGURE 1:**
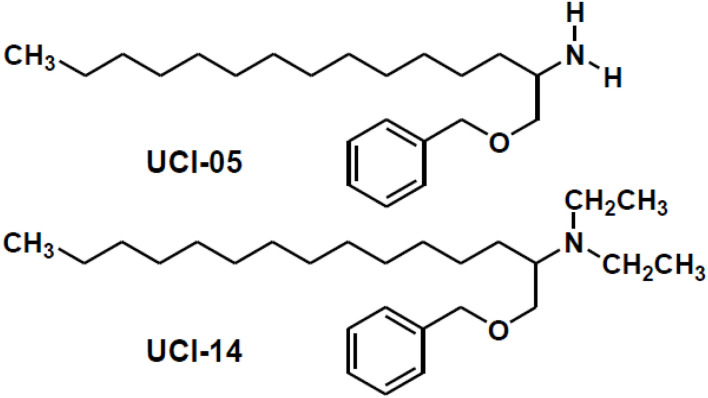
Structures of compounds used in this study.

New antitubercular compounds designated UCIs were synthesized as ethambutol (EMB) and sphingosine hybrids; UCI is an acronym to honor the institutions where these compounds were designed, produced, and tested: The **U**niversity of Salamaca, Pharmacy School, the **C**entro de Investigación Biomédica del Noreste, Mexican Institute of Social Security and the **I**nstitute of Medical Sciences and Nutrition, “Salvador Zubirán.” In particular, UCI-05 and UCI-14 (Figure 1) have shown good *in vitro* and *in vivo* activity against first line antitubercular drug susceptible and MDR strains, with minimal acute and subchronic toxicity in BALB/c mice (PCT EP 2007062381, WO 2008059014 A2) (Del Olmo et al., 2009; Olmo et al., 2016). Nevertheless, the mechanisms of action of UCI compounds on *M. tuberculosis* remain unknown. Antibacterial drug activity varies depending upon levels of resistance. It is essential to understand the mechanisms of action in order to select effective therapeutics (Fu and Shinnick, 2007). The study of drug-induced changes of gene expression profiles at the transcriptional level may generate new insights regarding genes related to the mechanism of action (Fu, 2006). We aimed to identify those genes modified by UCI-05 and UCI-14 exposure to acquire relevant information in the mechanism of action of these compounds in drug-sensitive and MDR strains of *M. tuberculosis*.

## Materials and Methods

### Cloning

*M. tuberculosis* clones were derived from the H37Rv strain (ATCC 27294) and from the clinical isolate CIBIN:UMF:15:99, which is resistant to all basic antitubercular medications [streptomycin (STR), isoniazid (INH), rifampicin (RIF), ethambutol (EMB), and pyrazinamide (PZA)] (Molina-Salinas et al., 2006). Frozen aliquots of each *M. tuberculosis* strain were thawed, inoculated on Lowenstein-Jensen (LJ, Merck, Darmstadt, Germany) slants and incubated at 37°C in a 5% CO_2_ atmosphere for 2–3 weeks. A loopful of the solid cultures was used to prepare homogenous suspensions of every strain that were adjusted to a turbidity equivalent to No. 1 McFarland standard (1.97 × 10^6^ UFC/mL in *M. tuberculosis* H37Rv cultures (Penuelas-Urquides et al., 2013b). Serial dilutions of all suspensions were performed (10^–1^ to 10^–8^) and drip-inoculated in Middlebrook 7H10 solid medium (Difco, Becton Dickinson, Le Pont de Claix, France) supplemented with 10% OADC enrichment (oleic acid, albumin, dextrose, and catalase, Becton Dickinson and Company, Sparks, MD, United States) to obtain isolated colonies. Plate cultures were incubated at 37°C in 5% CO_2_ atmosphere for 3 weeks; three isolated colonies from every strain were selected and inoculated separately in liquid media Middlebrook 7H9 broth (Difco, Becton Dickinson, Le Pont de Claix, France) supplemented with 10% OADC and incubated at 37°C in 5% CO_2_ atmosphere for 3 weeks. Finally, liquid cultures of clones were inoculated in Lowenstein-Jensen (LJ, Merck, Darmstadt, Germany) slants to obtain enough biomass for subsequent analysis. All experiments with *M. tuberculosis* were conducted considering biosafety practices for BSL-3 laboratories by authorized and trained researchers. This project was revised and approved by the institutional scientific committee.

### First-Line Drug Susceptibility Testing

Clones derived from H37Rv and CIBIN:UMF:15:99 strains were evaluated by drug susceptibility using the BACTEC MGIT 960 system (Becton Dickinson and Company, Sparks, MD, United States) as previously described (Penuelas-Urquides et al., 2013a) using the following critical concentrations of drugs: STR, 1.0 μg/mL; INH, 0.1 μg/mL; RIF, 1.0 μg/mL; EMB, 5.0 μg/mL, and PZA, 150 μg/mL. At concentration of 100 μg/mL variability in the determination of PZA resistance for clinical isolates of *M. tuberculosis* had been observed (Cheng et al., 2000). In our study, the H37Rv strain and clones were capable to growth at 100 μg/mL in the Bactec 960 system, but they do not at 150 μg/mL. Our results were confirmed using the pyrazinamidase test.

### Pyrazinamidase Test

A fresh inoculum of *M. tuberculosis* grown in solid LJ medium was placed on the surface of the pyrazinamidase medium, composed of Bacto Dubos medium (Difco, Becton Dickinson), 0.8 mM PZA (Sigma Aldrich, Steinheim, Germany), sodium pyruvate and bacteriological agar (Bioxon, Becton Dickinson, Estado de Mexico, Mexico). Cultures were incubated at 37°C for 4 days; then 1 mL of 1% ferrous ammonium sulfate solution was added to each culture, and tubes were incubated at room temperature for 30 min. The appearance of a pink band revealed the enzymatic hydrolysis of PZA to pyrazinoic acid and the culture was considered as positive to pyrazinamidase activity and therefore, susceptible to PZA. Cultures with negative results were repeated after 7 days of incubation to confirm resistance to PZA. *M. avium* and *M. bovis* cultures were included as positive and negative controls for pyrazinamidase activity, respectively.

### DNA Isolation

Mycobacterial DNA was isolated using the cetyltrimethyl ammonium bromide (CTAB) method as previously described (Penuelas-Urquides et al., 2013a). Briefly, a suspension of each *M. tuberculosis* clone was prepared and inactivated at 80°C for 1.0 h. After cooling, 50 μL of lysozyme [10 mg/mL] (Invitrogen, Carlsbad, CA. United States), 70 μL of 10% sodium dodecyl sulfate (US Biological, Swampscott, MA. United States), and 6 μL of proteinase K [10 mg/mL] (Invitrogen, Carlsbad, CA. United States) were added. Samples were incubated at 65°C for 10 min, followed by the sequential addition of 100 μL of 5.0 M NaCl, 100 μL solution of 10% CTAB, and 0.7 M NaCl. Samples were incubated at 65°C for 10 min and 700 μL of chloroform:isoamyl alcohol was added (24:1). The supernatant was recovered, and the DNA contained in this fraction was precipitated with isopropanol, ethanol-washed and resuspended in Tris-EDTA buffer. DNA purity and concentration were determined by spectrophotometry at a ratio of wavelength of 260/280 nm using a NanoDrop 2000 spectrophotometer (Thermo Fisher Scientific, Wilmington, DE, United States), and the integrity was visualized in 1% agarose gels stained with 0.5 μL of ethidium bromide [10 mg/mL] (Invitrogen, Carlsbad, CA. United States).

### Clone Genotyping

DNA regions of interest in drug-sensitive and MDR strains and clones were evaluated by space oligo typing (spoligotyping) (Kamerbeek et al., 1997) and restriction fragment length polymorphism (RFLP)-IS*6110* (van Embden et al., 1993) as previously reported. Spoligotyping binary code and clades of strains and clones were based on the database of genotypic markers for *M. tuberculosis*^1^ (Demay et al., 2012).

### Bacterial Growth Rate

The growth curves in the absence and presence of the combination of 4 antitubercular drugs were performed for the phenotypic characterization of all *M. tuberculosis* clones. Drug concentrations used were based on the critical concentrations suggested for the Bactec 460 system (Said-Fernandez et al., 2006) as follows: STR 2.0 μg/mL, INH 0.1 μg/mL, RIF 2.0 μg/mL and EMB 2.5 μg/mL. Mycobacterial clones were inoculated in Middlebrook 7H9 liquid media supplemented with 10% OADC and incubated at 37°C in 5% CO_2_ atmosphere in constant shaking at 300 rpm. Turbidity measurements were carried out in McFarland units every 3 days for 27 days using a nephelometer (ATB 1550, BioMérieux, France).

### Antitubercular Activity

The evaluation of *in vitro* antitubercular activities of UCI-05 and UCI-14 was performed using the Microplate Alamar Blue Assay (MABA) as previously reported (Molina-Salinas et al., 2006). Briefly, UCI-05 and UCI-14 were prepared at a concentration of 1.0 mg/mL in 100% DMSO. Two-step serial dilutions from 10.00 to 0.31 μg/mL of each drug were assayed in triplicate and the MICs of UCI-05 and UCI-14 were determined as reported (Molina-Salinas et al., 2006). EMB was used as a control in MABAs performed for all clones; in addition, RIF was used as a control for MABAs of the H37Rv strain and clones using final concentrations from 0.06 to 2.00 μg/mL and ofloxacin was used as control for MABAs of CIBIN:UMF:15:99 isolate and clones using final concentrations from 0.50 to 16.00 μg/mL. H37Rv is sensitive to the two drugs used as control while the CIBIN:UMF:15:99 is ofloxacin sensitive and rifampicin resistant.

### Drug Treatment of Mycobacterial Cultures

One loopful of each *M. tuberculosis* clone grown on Lowenstein-Jensen slants was transferred and suspended into 3.0 mL Middlebrook 7H9 medium, supplemented with 10% OADC and incubated at 37°C in a 5% CO_2_ atmosphere, until they reached a turbidity equivalent to 1.0 McFarland standard. An aliquot of 1.0 mL was transferred to a screw-capped polypropylene conical bottom tube (Corning, New York, United States) containing 10 mL Middlebrook 7H9 medium supplemented with 10% OADC and incubated at 37°C in 5% CO_2_ atmosphere with constant shaking (at 300 rpm) until mycobacteria reached growth log phase [and a turbidity equivalent to 3.0–4.0 McFarland standard (Penuelas-Urquides et al., 2013b)]. Then, each clone culture was treated with UCI-05, UCI-14 (at a final concentration equivalent to their respective MICs) or 0.1% DMSO (as control) and incubated under the same conditions for additional 4 h.

### Clone Total RNA Isolation

Total RNA was extracted from log-phase treated cultures as previously described (Penuelas-Urquides et al., 2013a), using TRIzol reagent (Invitrogen, Carlsbad, CA. United States) and the Fast Prep instrument (MP Biomedicals, Solon, OH, United States). Briefly, 10 mL liquid mycobacterial cultures were centrifuged for 20 min at 3,390 rpm at 4°C; mycobacterial pellets were dissolved in 0.8 mL of TRIzol and transferred to Fast Prep tubes containing Lysing Matrix B (MP Biomedicals, Solon, OH. United States). After three cycles in the Fast Prep instrument at 6 m/s for 20 s, chloroform and isopropyl alcohol were added and an ethanol wash was performed. Total RNA, resuspended in Tris-EDTA buffer, was treated with DNase I (Invitrogen, Carlsbad, CA, United States). NanoDrop spectrophotometry was used to evaluate the purity and concentrations of RNA, and its integrity was observed in 1% agarose gels stained with 0.5 μL of ethidium bromide [10 mg/mL].

### Microarray Hybridization Procedure and Data Analysis

Affymetrix TB-All antisense microarrays were used for gene expression analysis as previously described (Penuelas-Urquides et al., 2013a). These chips include the complete genome sequence of the H37Rv reference strain and interrogates 6,021 targets simultaneously. Microarray data was pre-processed using the MAS 5.0 algorithm (Irizarry et al., 2003). Raw intensity values were background-corrected, log2-transformed, and then quantile-normalized. A linear model using the limma package (Smyth, 2004) was fit to the normalized data to identify differential expression. The selection of differentially expressed genes was based according to the following parameters: a log fold-change ≥ 1.0 combined with a B-statistics greater than zero. That is to say, the parameter considered as a real modification of gene expression in the presence of each UCI was equivalent to a difference of least twofold with respect to the correspondent untreated clone either over- or under-expression and a probability of modification > 50%. The datasets presented in this study can be found in Gene Expression Omnibus (GEO) at https://www.ncbi.nlm.nih.gov/geo/query/acc.cgi?acc=GSE184172.

### Reverse Transcription and Real-Time PCR Assays

The same RNA preparation used for microarray analysis was used for real-time PCR assays. As previously described (Penuelas-Urquides et al., 2013a), the synthesis of cDNA was carried out from 2 μg of total RNA using M-MLV reverse transcriptase and random primers (Invitrogen, Carlsbad, CA. United States) according to the manufacturer’s instructions. The functionality of the cDNA was confirmed by the amplification of the constitutive *rrs* gene, which encodes the ribosomal RNA 16S subunit, and was visualized on 1% agarose gels stained with ethidium bromide.

### Validation of Microarray Data

Microarray data were validated by analyzing the expression of 17 genes differentially expressed by RT-qPCR with primers and TaqMan probes (Table 1) using the 7500 Fast Real Time PCR System (Applied Biosystems, Foster City, CA, United States), 3.0 μL of cDNA was used as template. The dynamic range curve was established from 1:16 to 1:1024 dilutions for each gene and the 1:128 dilution was selected in each case. Technical triplicates, negative template controls and *rrs* expression (as endogenous control) were included in parallel in all the assays. The analysis was performed with the comparative method of the threshold values; this method determines the effect of the experimental treatment on the expression of a candidate internal control gene (Livak and Schmittgen, 2001).

**TABLE 1:** Primers and TaqMan probes for *M. tuberculosis* genes used in this study.

Gene	Primers (5′–3′)	Probe (FAM 5′–3′ NFQ)
*desA2*	F-TCG AGT CCA ACA CGT GAT GAA G, R-CGC TCG TAG AAC GCC ATG TA	CTG CGT GTA CTT CTC G
*esxG*	F-CGG CTC AGG CGT TTC AC, R-CCG CCG CCA CAA ACC	CCG CCG ACG ACT CC
*esxH*	F-CAG GCC GCG TTG CA, R-CTG CCA CGC CTG ATA CGT	CCC TGC CAC GCA CTC
*esxI*	F-GAC CGC GAG TGA CTT TTG G, R-GCT CGT AGA TCA CCT GGA AGT TAC	CAG GCC GCC GAA CCG
*gltA1*	F-CGG TTA TGC GCA GAA CTT CCT, R-CAC GAC GGC GGT TTC G	ACC TCC CCG AAG CAC
*groES*	F-GGA GGG CAC CGT CGT T, R-GTC ACC CTC CGC AAC GT	CTC GCC GTC CTC GTC C
*hspX*	F-GTC CGC GAT GGT CAG CT, R-CGA AGG AAC CGT ACG CGA AT	ACC GTC GAA GTC CTT C
*infA*	F-CCT GCC CAA TGC CAT GTT C, R-ACG CAT CTT GCC GCT GAT	CCT TGT GGC CGT TCT C
*iniB*	F-GCT AGC CAG ATC GGT GTC T, R-CAG GCC GCT GAC ATT GC	CCG CCG AGC CCA CCA C
*lldD2*	F-TCG ACG CGA TGG GAC AC, R-GGC CAG CGA TCC AGT GAA	TTG ACC ACC GAA CCG C
*lprQ*	F-GCG TCC TCG GGA GTA CTA C, R-CCG AAC GGC AGC CCA T	TCG ACG CCA AGC TGT
*mmaA4*	F-GAC CGG ATT GTG TCG ATC GAA, R-CGC TTG AAG AAG TCG TCG TAG TT	TCG TGC CCG AAG TGC
*nrdH*	F-AAG GTT GAT ATC AGC CTG GAT TCC, R-CGA CGG GTG CTT GTA GGT AA	CCC AGC GCC ATC ACG T
*rpmI*	F-CGG TAC CGG CAA GAT CGT, R-CGG GTG CTC GGC TTG T	ACG GTT GGC CTT CTG C
*rpsO*	F-ATC GCG TTG CTG ACC AAA C, R-CGA ATG ATG GTC GTG CTT GTG	CAT CGC CGA CCT CAC C
*tatA*	F-CGC TGT CGT GGT GAT CGT, R-TCG GAC TTA AAG ATT CGC AAT GAC T	CTT GGC ACC GAA CAA C
*whiB1*	F-GCC CGG TCA CCA CAG A, R-CAG ACG CCC GAG TCC TG	CTG GGC ACT GAA TAC C
*rrs*	F-GGG TCT CTG GGC AGT AAC TG, R-GTG GAC TAC CAG GGT ATC TAA TCC T	TCG CTC CCC ACG CTT T

*NFQ, Non-fluorescent quencher.*

### Statistical Analysis

Mean values ± standard deviations (SD) from biological and technical replicates of quantitative PCR results were graphed. Statistical comparisons were made using the Student’s *t*-test and significance was set at *p* ≤ 0.05. All analyses were conducted using STATA version 8.1 (Stata Corporation, College Station, TX, United States).

## Results

*M. tuberculosis* clones were derived from the H37Rv strain (ATCC 27294), which is sensitive to first-line antitubercular drugs, and from the MDR clinical isolate- CIBIN:UMF:15:99. All three clones derived from H37Rv and CIBIN:UMF:15:99 were genotyped. The H37Rv strain and its three clones lacked spacers 20 and 21 while the CIBIN:UMF:15:99 strain and its three clones lacked spacer 18. All parent strains and clones of both H37Rv and CIBIN:UMF:15:99 lacked spacers 33–36 (Supplementary Figure 1). The spoligotype of H37Rv and its three clones matched the H37Rv pattern registered in the Database of Genotyping Markers for *M. tuberculosis*. In contrast, the spoligotype of CIBIN:UMF:15:99 and its three clones matched the X1 clade, according to the aforementioned database. RFLP-IS*6110* typing assays revealed that the H37Rv strain and its clones displayed 15 copies of the IS*6110* insertion element, compared to only 4 copies in the CIBIN:UMF:15:99 isolate and its clones (Supplementary Figure 1).

**FIGURE 2:**
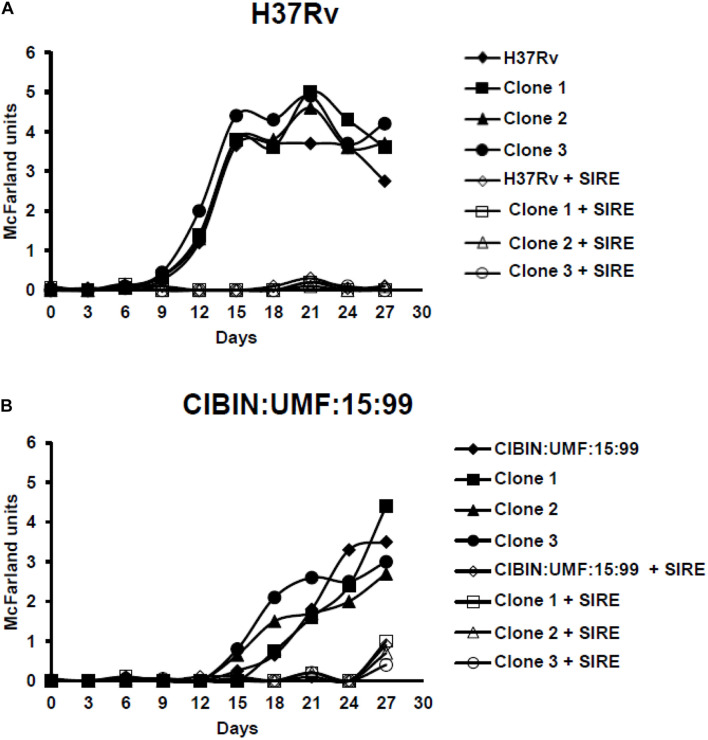
Kinetic growth curves of drug-sensitive H37Rv **(A)** and MDR CIBIN:UMF:15:99 **(B)** strains and clones in absence and presence of streptomycin (S), isoniazid (I), rifampicin (R), and ethambutol (E). Critical concentrations of each drug were used (S, 2 μg/mL; I, 0.1 μg/mL; R, 2 μg/mL; E, 2.5 μg/mL). Measurements were taken every 3 days by McFarland turbidity. Filled symbols represent cultures of the H37Rv and CIBIN:UMF:15:99 strains and clones in the absence of drugs; empty symbols represent cultures of H37Rv and CIBIN:UMF:15:99 strains and clones in the presence of drugs. SIRE, Streptomycin, Isoniazid, Rifampicin, Ethambutol.

The phenotype of the strains and their respective clones were characterized by first-line drug susceptibility testing. H37Rv clones were sensitive to all 5 antitubercular drugs (STR, INH, RIF, EMB and PZA) while CIBIN:UMF:15:99 clones were resistant. Pyrazinamidase assays confirmed the susceptibility of H37Rv strain and clones, and the resistance of CIBIN:UMF:15:99 strains and clones to PZA (data not shown). In addition, for the phenotypic characterization of clones, kinetic growth curves were performed in the absence and presence of the combination of four antitubercular drugs (STR 2.0 μg/mL, INH 0.1 μg/mL, RIF 2.0 μg/mL, and EMB 2.5 μg/mL); PZA was not included because it acts in acidic environment (Zhang et al., 2003). Clones of H37Rv grew only in the absence of antitubercular drugs. In contrast, CIBIN:UMF:15:99 clones grew both in the absence and presence of the four drug combination, although growth in the presence of drugs was not observed until the 27th day of incubation (Figure 2).

The antitubercular activity of UCIs was assessed through Microplate Alamar Blue Assay. Table 2 shows that the minimum inhibitory concentration (MIC) of UCI-05 against CIBIN:UMF:15:99 and its three clones (7.2 μM) was lower than that for H37Rv and its clones (14.4 μM). However, the MIC of UCI-14 was identical against H37Rv and CIBIN:UMF:15:99 and their clones (12.4 μM), except for a higher MIC for the H37Rv-clone 1 (24.8 μM).

**TABLE 2:** Determination of the minimum inhibitory concentrations (MICs) of UCI compounds against sensitive (H37Rv) and multi-drug resistant (CIBIN:UMF:15:99) strains and clones of *M. tuberculosis*.

Strain/Clone	MIC (μM)		
	UCI-05	UCI-14	EMB
H37Rv strain	14.4	12.4	12.2
H37Rv Clone-1	14.4	24.8	12.2
H37Rv Clone-2	14.4	12.4	12.2
H37Rv Clone-3	14.4	12.4	12.2
CIBIN:UMF:15:99 strain	7.2	12.4	48.9
CIBIN:UMF:15:99 Clone-1	7.2	12.4	48.9
CIBIN:UMF:15:99 Clone-2	7.2	12.4	48.9
CIBIN:UMF:15:99 Clone-3	7.2	12.4	48.9

**FIGURE 3:**
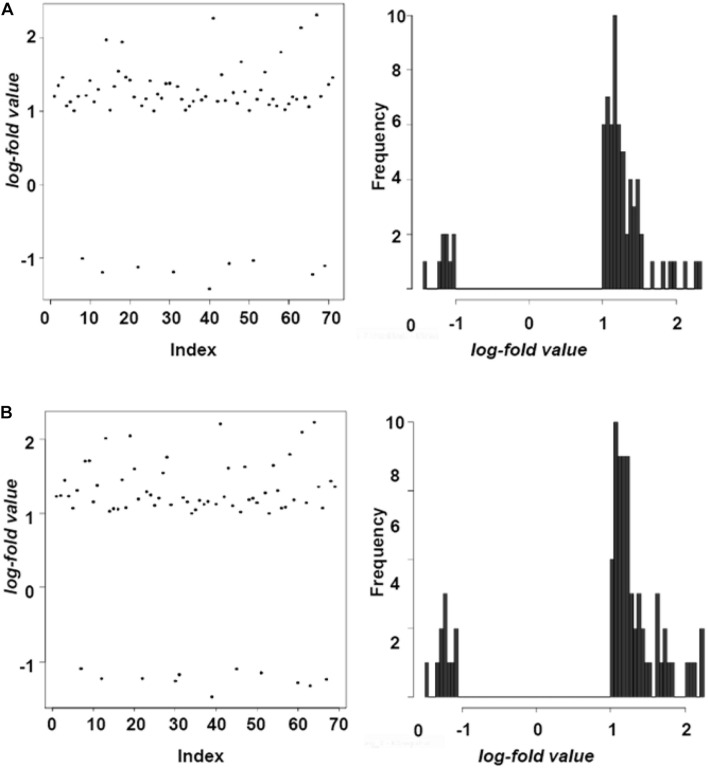
Differential gene expression in H37Rv clone exposed to UCI compounds. Differentially expressed genes are shown with points in the left panel and with bars in the right panel. A value ≥ 1 log fold change shows induced genes and ≤1 shows repressed genes. Representative assays of altered expression in H37Rv clone 2 by **(A)** UCI-05 where 62 regions were over-expressed, and 9 regions were down-regulated; and by **(B)** UCI-14 where 58 regions were over-expressed, and 11 regions were down-regulated.

For the evaluation of gene expression profiles of clones exposed to UCI compounds, total RNA samples isolated from *M. tuberculosis* cultures treated with UCI-05 and UCI-14 were analyzed by Affymetrix TB-All antisense microarrays. Figure 3 and Supplementary Figure 2 show that in the H37Rv clone 2 treated with UCI-05, gene expression changed in 71 regions that included 22 annotated genes, 29 hypothetical proteins, 13 intergenic regions, and 7 tRNAs. In the same clone treated with UCI-14, differential gene expression was observed in 69 regions that included 19 annotated genes, 26 hypothetical proteins, 17 intergenic regions and 7 tRNAs. In H37Rv clone 3 treated with UCI-14, 56 genetic regions showed differential expression; 25 of these were annotated genes, 12 hypothetical proteins, 14 intergenic regions, and 5 tRNAs (Supplementary Material 1).

We selected 17 genes with altered expression detected by microarrays (Table 3) and compared these data to those obtained by RT-qPCR assays and found a concordance of 28%. The 17 selected genes were analyzed by RT-qPCR in all H37Rv and CIBIN:UMF:15:99 clones.

**TABLE 3:** Genes differentially expressed by *M. tuberculosis* drug-sensitive (H37Rv) and MDR (CIBIN:UMF:15:99) clones treated with UCI-05 or UCI-14.

Gene	Description	Functional category
*desA2*	Possible acyl-desaturase DesA2	Lipid metabolism
*esxG*	ESAT-6 like protein EsxG	Cell Wall and cell processes
*esxH*	Low molecular weight protein antigen 7 EsxH	Cell Wall and cell processes
*esxI*	Putative ESAT-6 like protein EsxI	Cell Wall and cell processes
*gltA1*	Probable citrate synthase GltA1	Intermediary metabolism and respiration
*groES*	10 kDa chaperonin GroES	Virulence, detoxification, adaptation
*hspX*	Heat shock protein HspX	Virulence, detoxification, adaptation
*infA*	Probable translation initiation factor IF-1	Information pathways
*iniB*	Isoniazid inductible gene protein IniB	Cell Wall and cell processes
*lldD2*	Possible L-lactate dehydrogenase (cytochrome) Lldd2	Intermediary metabolism and respiration
*lprQ*	Probable conserved lipoprotein LprQ	Cell Wall and cell processes
*mmaA4*	Methoxy mycolic acid synthase 4 MmaA4	Lipid metabolism
*nrdH*	Glutaredoxin-like protein NrdH	Information pathways
*rpmI*	Probable 50 s ribosomal protein L35	Information pathways
*rpsO*	Probable 30 s ribosomal protein S15	Information pathways
*tatA*	Probable sec-independent protein translocase membrane-bound protein TatA	Cell Wall and cell processes
*whiB1*	Probable transcriptional regulatory protein WhiB-like WhiB1	Regulatory proteins

**FIGURE 4:**
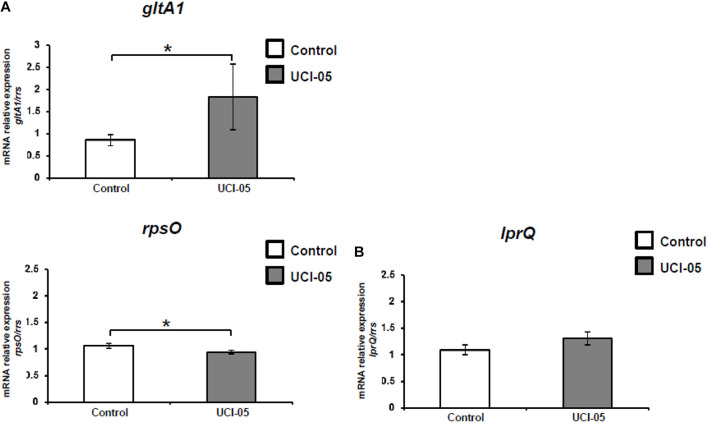
RT-qPCR assays of genes differentially expressed in H37Rv **(A)** and CIBIN:UMF:15:99 **(B)** clones after UCI-05 exposure. Errors bars show standard deviations of the mean of three biological assays (each one with technical triplicates). *P < 0.05. Data were analyzed with Student’s *t*-test.

Figure 4 shows that H37Rv clones treated with UCI-05 showed an over-expression average of 2.5-fold in *gltA1* (*P* = 0.05) while the expression of *rpsO* was down-regulated by 11.08% (*P* = 0.02). In CIBIN:UMF:15:99-clones treated with UCI-05, *lprQ* gene showed a trend of over-expression (20%, *P* = 0.06).

**FIGURE 5:**
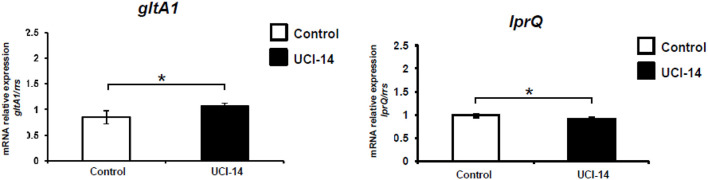
RT-qPCR assays of differentially expressed genes in H37Rv clones after UCI-14 exposure. Errors bars show standard deviations of the mean of three biological assays (each performed in technical triplicates). *P < 0.05. Data were analyzed with Student’s *t*-test.

In contrast, the average relative gene expression in H37Rv clones treated with UCI-14 showed a 26% over-expression of *gltA1* (*P* = 0.04) as well as 8.08% down-regulation of *lprQ* (*P* = 0.02) (Figure 5).

**FIGURE 6:**
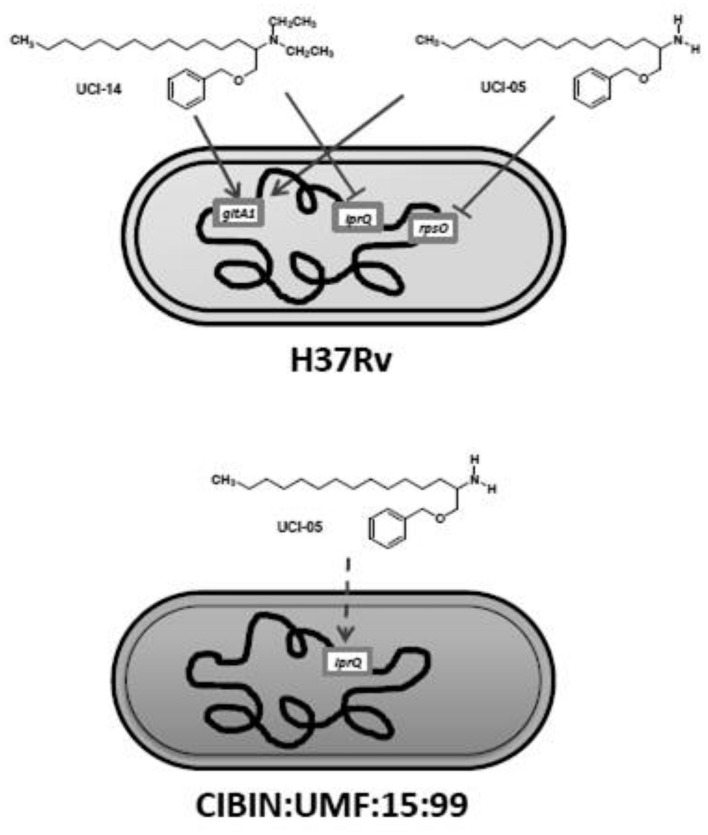
Hypothetical model of target genes affected by UCI-05 and UCI-14 in drug sensitive (H37Rv) and MDR (CIBIN:UMF:15:99) *M. tuberculosis*. Both UCI-05 and UCI-14 induced *gltA1* expression in H37Rv strain, whereas UCI-05 and UCI-14 repressed *rpsO* and *lprQ* expression, respectively, in H37Rv strain. UCI-05 caused an induction trend for *lprQ* expression in MDR strain. Continuous arrows mean gene induction modified significantly; dashed arrow represents a trend of gene induction; inhibition arrows represent that the compounds significantly down-regulated target genes.

CIBIN:UMF:15:99 clones exposed to UCI-14 did not show differences in the expression of the 17 selected genes by RT-qPCR. All comparisons were performed with the same clones treated with 0.1% DMSO as control. Figure 6 shows a hypothetical model of target genes affected by UCI-05 and UCI-14 in drug sensitive (H37Rv) and MDR (CIBIN:UMF:15:99) *M. tuberculosis* strains.

## Discussion

Wide phenotypic variations can occur in isogenic bacterial population (Longo and Hasty, 2006). Factors that contribute to this diversity are noise and random fluctuations of gene expression that may mask the behavior of each individual cell, affecting the identification of biologically important cellular processes (Raser and O’Shea, 2005; Longo and Hasty, 2006). This heterogeneity has been also described in the *M. tuberculosis* H37Rv strain (Andreu and Gibert, 2008). To analyze gene expression in independent bacterial populations treated with new antitubercular compounds, we isolated clones from the drug-sensitive strain *M. tuberculosis* H37Rv, and from the clinical MDR isolate CIBIN:UMF:15:99.

Genotypic characterization of *M. tuberculosis* clones using spoligotyping and RFLP-IS*6110* showed that H37Rv and CIBIN:UMF:15:99 have different patterns and belong to different clades. All clones shared identical band patterns with their parental strains preserving the original profiles and suitability for gene expression studies. However, *M. tuberculosis* H37Rv clones had different MICs for UCI-14; clone 1 had a MIC of 24.8 μM whereas clones 2 and 3 had a MIC of 12.4 μM. These results suggest some variability between individuals of the same strain.

CIBIN:UMF:15:99 and its clones grew slower in liquid medium than H37Rv and its clones. It has been reported that MDR strains have a longer lag phase due to their reduced ability to adapt to new growth conditions. This may be due to mutations in genes such as *katG* and *rpoB*, which encode biologically important enzymes that may impact bacterial fitness (Mariam et al., 2004; von Groll et al., 2010b). Results from our working group confirmed that CIBIN:UMF:15:99 has mutations in both *katG* and *rpoB* genes (Ser315Thr, Ser531Leu, respectively, unpublished results and personal communication). In our results, the MDR clones showed a longer lag phase (12–15 days) than sensitive clones (9 days). In addition, it has been reported that the growth rates of MDR strains are lower than those drug-sensitive strains (von Groll et al., 2010a), and can normalize once the strains are adapted to a new environment, probably because of the generation of compensatory mutations (O’Sullivan et al., 2010). We observed that H37Rv cultures reached a turbidity equivalent to 4.0–5.0 McFarland standards in the logarithmic growth phase, while the CIBIN:UMF:15:99 clones reached a turbidity of 3.5–4.5 McFarland standards.

Once the growth kinetics of the clones were determined, we defined the appropriate time to perform UCIs exposures. The logarithmic growth phase allows the observation of altered expression of at least half of the genes, which are active during this phase (Fu and Fu-Liu, 2007); in contrast, during the stationary growth phase, a high percentage of genes are repressed (Voskuil et al., 2004).

The MICs of UCI compounds were reported in 2009 (as compound **3** for UCI-05 and compound **4b** for UCI-14); UCI-05 had a MIC of 3.6 μM for H37Rv and CIBIN:UMF:15:99 strains and UCI-14 had a MIC of 3.1 μM for both strains (Del Olmo et al., 2009). Inconsistencies between the MICs reported previously and those observed in our results may be due to differences between the batches of UCI compounds and/or different circumstances and conditions for the respective bioassays. In our results, EMB was slightly more potent than UCIs in the sensitive strain and clones but importantly UCI MICs in the MDR strain and clones were markedly inferior than EMB and also inferior to previously reported MICs of first-line drugs INH (22.8 μM) and RIF (122 μM) (Molina-Salinas et al., 2006) against CIBIN:UMF:15:99.

In gene expression analysis, RT-qPCR showed that exposure to UCI-05 resulted in a statistically significant over-expression of *gltA1* and a statistically significant down-regulation of *rpsO* in the sensitive H37Rv clones, and a trend of over-expression of *lprQ* in CIBIN:UMF:15:99 clones. In contrast, H37Rv clones exposed to UCI-14 showed a statistically significant over-expression of *gltA1* and a statistically significant down-regulation of *lprQ*. Over-expression of *gltA1* has been reported when the H37Rv strain is exposed to sub-inhibitory concentrations of plumbagin (Ye et al., 2011). This gene encodes citrate synthase I, which catalyzes the conversion of oxaloacetate plus acetyl-CoA into citrate and coenzyme A in the glyoxylate cycle (Reddy et al., 2009) and the conversion of oxaloacetate plus propionyl-CoA into (2S, 3S)-2-methylcitrate in the methylcitrate cycle; both processes are involved in the central carbon metabolism of *M. tuberculosis* (Reddy et al., 2009; Rhee et al., 2011). In particular, the glyoxylate cycle metabolizes acetyl-CoA while the methylcitrate cycle metabolizes propionyl-CoA (Munoz-Elias et al., 2006), both generated during β-oxidation of fatty acids (Rhee et al., 2011). Based upon the results obtained in this study, we suggest that over-expression of *gltA1* during UCI-05 and UCI-14 exposure could be a response mechanism of *M. tuberculosis* to prevent the intracellular accumulation of UCI compounds to inhibitory levels; lipid degradation pathways may be upregulated (Munoz-Elias et al., 2006; Upton and McKinney, 2007) because these compounds contained a lipid chain derived from dihydrosphingosine (Del Olmo et al., 2009).

Drug-sensitive clones treated with UCI-05 showed a statistically significant down-regulation of *rpsO*. This gene has been reported as one of the 100 most expressed genes during log-phase growth in *M. tuberculosis* (Fu and Fu-Liu, 2007); it encodes for the ribosomal protein S15, which is a component of the 16S ribosomal subunit in *Bacillus stearothermophilus*. Its function is to couple the ribosome to mRNA targets independently of their sequence (Scott and Williamson, 2005). The down-regulation of *rpsO* during exposure to UCI-05 observed in this study suggests that translation could be affected in *M. tuberculosis* as a stress response.

MDR clones showed a different gene expression profile during UCI exposure, featuring over-expression of *lprQ* when treated with UCI-05, while drug-sensitive clones showed a down-regulation of this gene when treated with UCI-14. The *lprQ* gene encodes a lipoprotein that belongs to the family ErfK/YbiS/YcfS/YnhG (Pfam PF03734) (Sutcliffe and Harrington, 2004). Lipoproteins have been identified as new drug targets in Gram positive bacteria (Hutchings et al., 2009). In *M. tuberculosis*, mutations in genes involved in lipoproteins synthesis decrease growth in macrophages during *in vitro* studies and attenuate their virulence (Sander et al., 2004). To date, the LprQ lipoprotein has not been fully characterized and its function remains unknown (Sutcliffe and Harrington, 2004; Reddy et al., 2009).

UCI compounds are EMB derivates. The over-expression of the efflux pump genes *Rv0194* and *Rv1273c* have been reported in response to EMB exposure in *M. tuberculosis* H37Rv (Garima et al., 2015), although neither of these genes showed differential expression after UCIs exposure.

Further experiments are necessary to confirm the participation of the modified genes, both in the H37Rv and CIBIN:UMF:15:99 strains, in response to UCIs. Some of these approaches include the generation of mutants for the identified genes, as well as exogenous genetic overexpression assays in the presence of UCIs. Furthermore, it is possible the gene expression inhibition using antisense sequences directed against transcriptional regulators of genes modified by UCIs.

## Conclusion

In conclusion, UCI-05 and UCI-14 increase *gltA1* expression in drug-sensitive *M. tuberculosis*, which could reflect an increased activity of glyoxylate and methylcitrate pathways. In addition, UCI-05 could alter translation in drug-sensitive strains by down-regulating *rpsO*. Furthermore, UCI-05 increases *lprQ* expression in MDR clones while UCI-14 represses this gene in sensitive clones, although the LprQ lipoprotein has not been fully characterized. Our findings present relevant data regarding the *M. tuberculosis* response to novel antitubercular compounds at the transcriptional level in sensitive and MDR strains. It is essential to study the mechanisms by which UCI-05 and UCI-14 alter the expression levels of *gltA1*, *rpsO*, and *lprQ*, in order to develop targeted and less toxic therapeutics.

## Data Availability Statement

The datasets presented in this study can be found in Gene Expression Omnibus (GEO) at https://www.ncbi.nlm.nih.gov/geo/query/acc.cgi?acc=GSE184172.

## Author Contributions

KP-U, BS-R, MBL, and SS-F were involved in the conception, article drafting, critical revision, and design of study. KP-U, GM-S, and PB-M were involved in the acquisition of laboratory data. EO and AS were involved in the acquisition of laboratory data of development, production, and characterization of UCI compounds. KP-U, MBL, and FC-T were involved in analysis of data and statistics. KP-U, MBL, BS-R, FC-T, GM-S, JC-G, PB-M, EO, AS, LG-E, LV-T, and SS-F were involved in the revision and approved the final version of the manuscript. All authors contributed to the article and approved the submitted version.

## Conflict of Interest

The authors declare that the research was conducted in the absence of any commercial or financial relationships that could be construed as a potential conflict of interest.

## Publisher’s Note

All claims expressed in this article are solely those of the authors and do not necessarily represent those of their affiliated organizations, or those of the publisher, the editors and the reviewers. Any product that may be evaluated in this article, or claim that may be made by its manufacturer, is not guaranteed or endorsed by the publisher.

## Footnotes

1
http://www.pasteur-guadeloupe.fr:8081/SITVIT_ONLINE/query
