# Supplementary material for: Two New Dihydrosphingosine Analogs Against Mycobacterium tuberculosis Affect gltA1, lprQ, and rpsO Expression
Source: Front Microbiol. 2021 Nov 3;12:742867. doi: 10.3389/fmicb.2021.742867 (PMC8595602; doi:10.3389/fmicb.2021.742867)
Supplement: Supplementary file 1 [file Data_Sheet_1.docx]

SUPPLEMENTARY MATERIAL


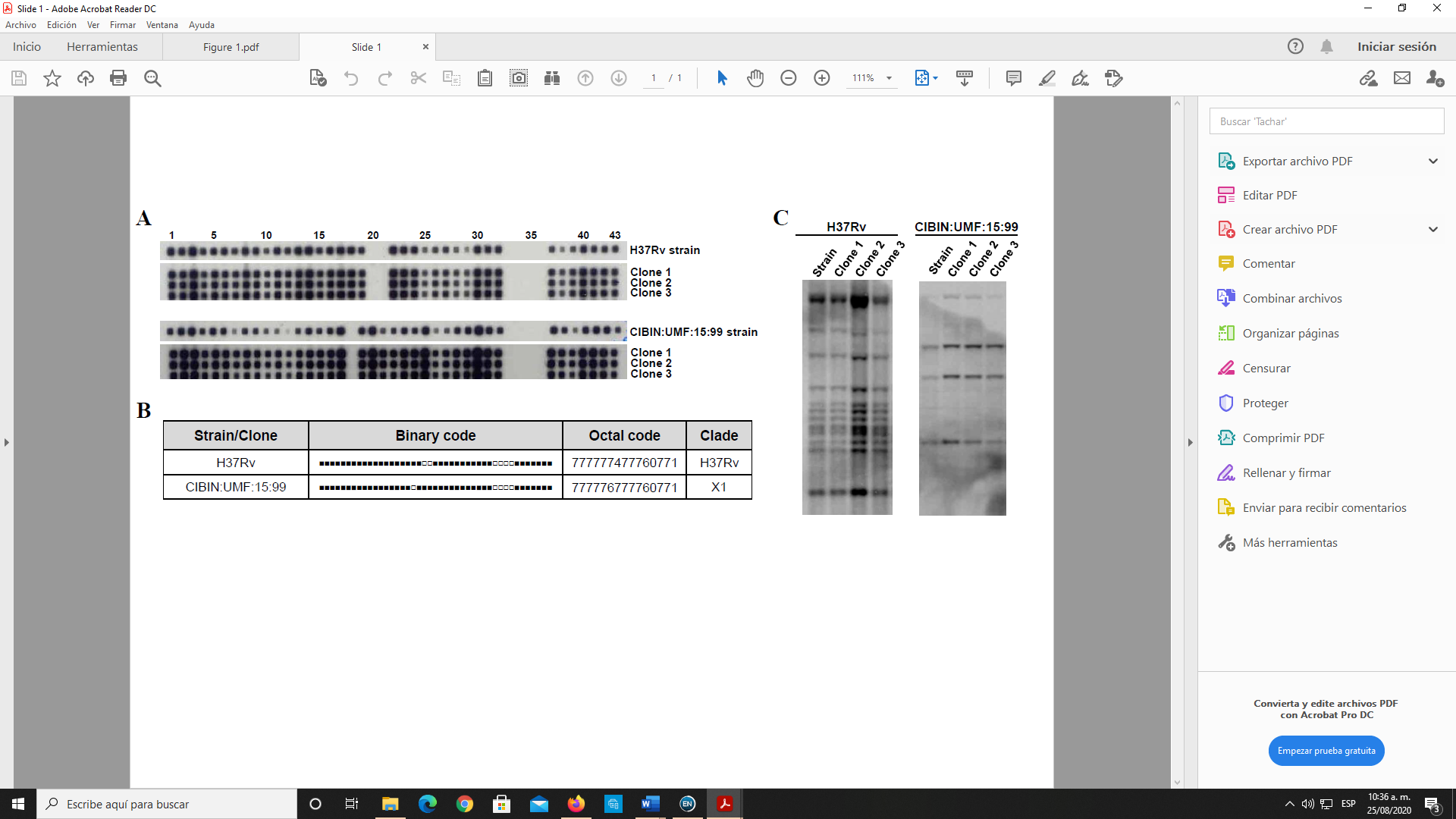
 **Figure S1.** Genetic characterization of drug-sensitive and multi-drug resistant (MDR) strains and clones of *M. tuberculosis*. **(A)** Spoligotyping disclosed that the H37Rv strain and clones lacked spacers 20 and 21; the CIBIN:UMF:15:99 strain and clones lacked spacer 18. All strains and clones lacked spacers 33 to 36. **(B)** Spoligotyping binary code and clades of strains and clones based on the database of genotypic markers for *M. tuberculosis* (see Materials and Methods section). **(C)** RFLP-IS*6110* assays. H37Rv strain and clones have 15 copies of IS*6110* element, while the CIBIN:UMF:15:99 strain and its clones have 4 copies.


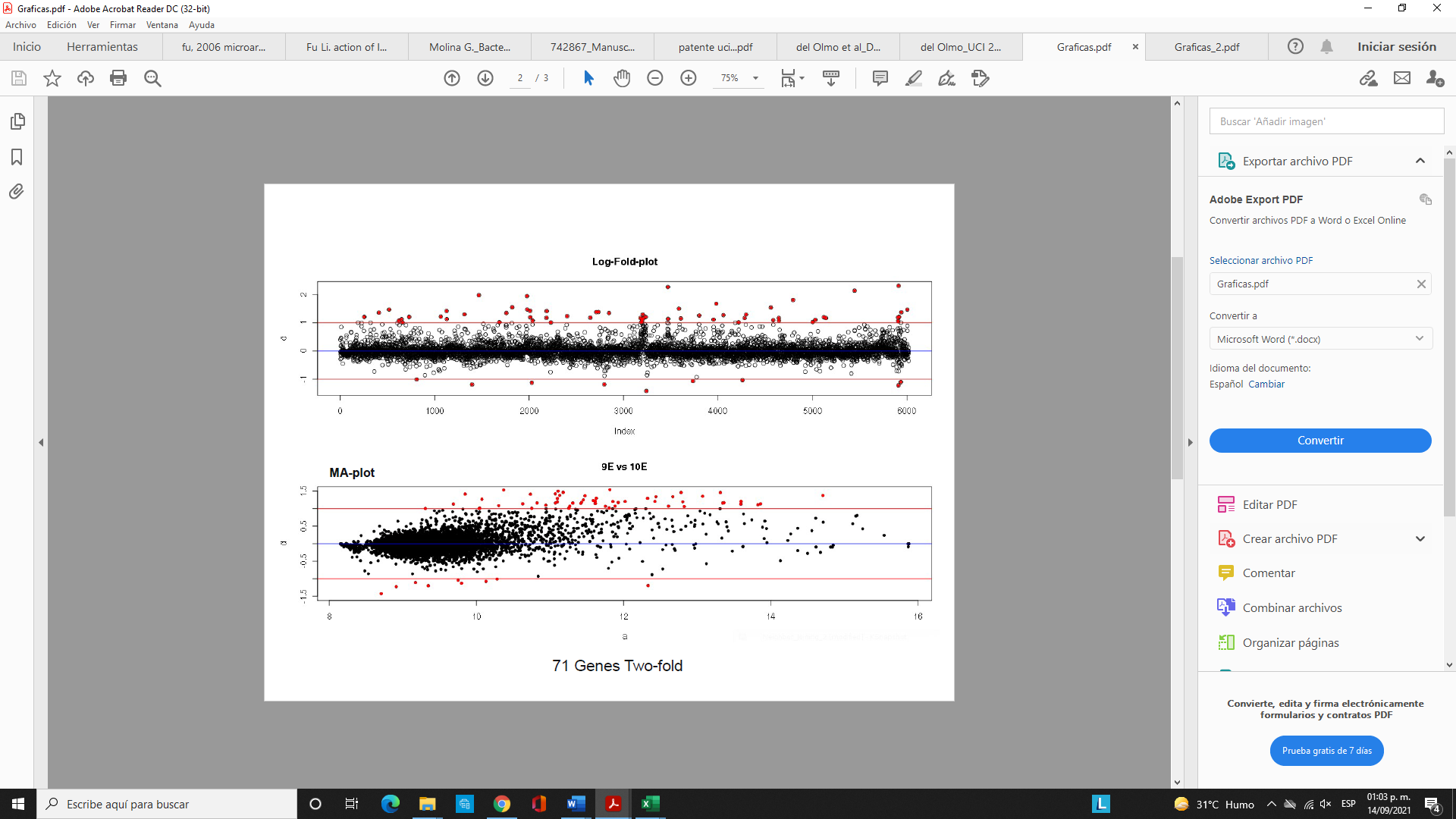

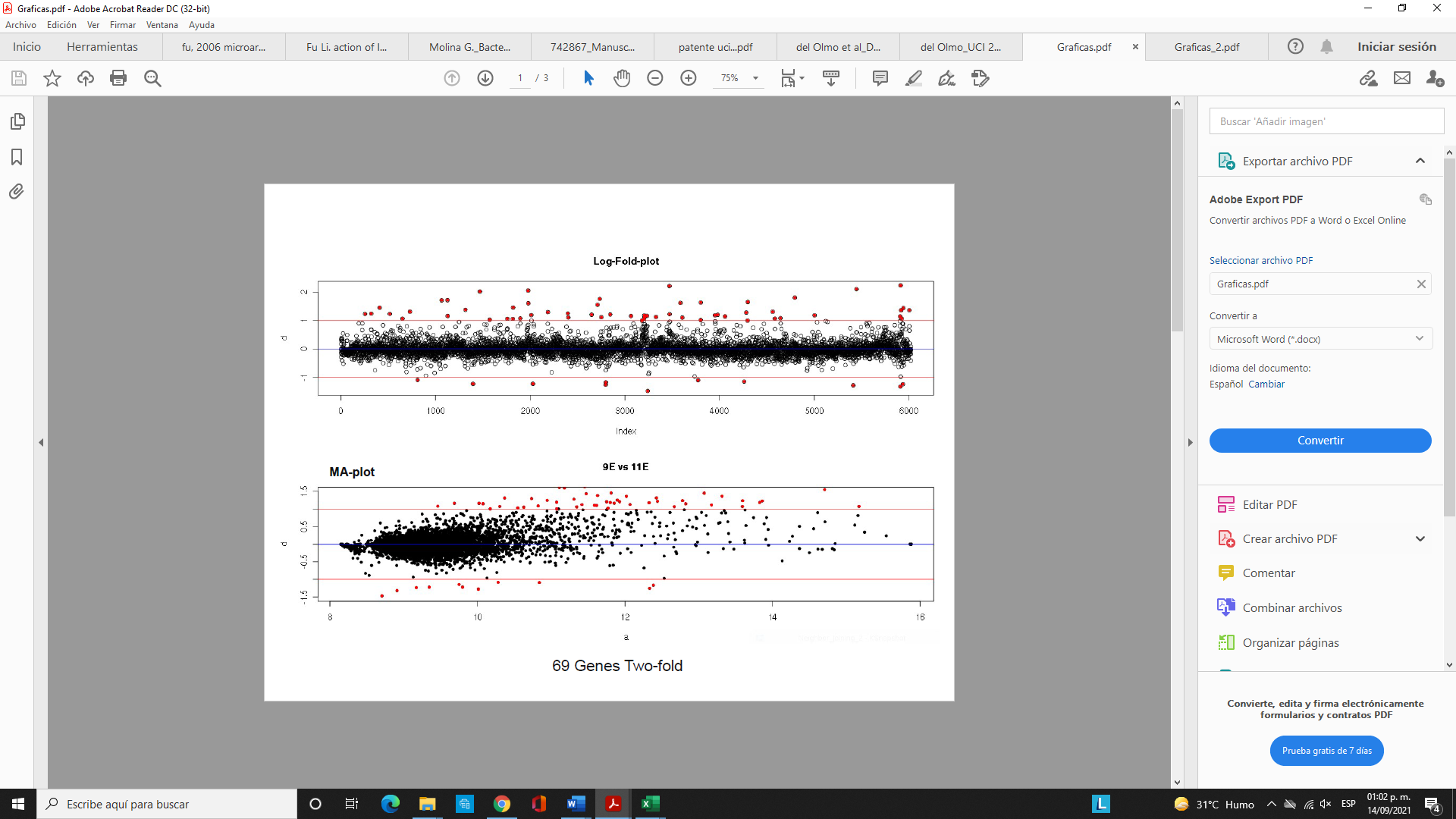


**A**

**B**

**Figure S2.** MA-plot showing differential gene expression in H37Rv clone exposed to UCI compounds. A value > 1 log fold change shows induced genes and < 1 shows repressed genes. Representative assays of altered expression in H37Rv clone 2 by **(A)** UCI-05 where 62 regions were over-expressed, and 9 regions were down-regulated; and by **(B)** UCI-14 where 58 regions were over-expressed, and 11 regions were down-regulated.

Supplementary Material 1**.** Data of gene expression of clones exposed to UCI compounds analyzed by Affymetrix TB-All antisense microarrays.

9E: H37Rv clone 2 treated with 0.1% DMSO; 10E: H37Rv clone 2 treated with UCI-05; 11E: H37Rv clone 2 treated with UCI-14; 12E: H37Rv clone 3 treated with 0.1% DMSO; 14E: H37Rv clone 3 treated with UCI-14.
